# Exploring fraity and sarcopenia in older adults admitted to acute medical unit, looking at prevalence, trajectory, and outcomes: A protocol testing the feasibility and acceptability of the TYSON study

**DOI:** 10.1371/journal.pone.0293650

**Published:** 2023-11-03

**Authors:** Vicky Kamwa, Thomas Jackson, Zaki Hassan-Smith, Elizabeth Sapey

**Affiliations:** 1 Department of Acute Medicine, Queen Elizabeth Hospital Birmingham, Birmingham, West Midlands, United Kingdom; 2 Birmingham Acute Care Research Group, Institute of Inflammation and Ageing, The University of Birmingham, Birmingham, West Midlands, United Kingdom; 3 Department of Geriatrics, Queen Elizabeth Hospital Birmingham, Birmingham, West Midlands, United Kingdom; 4 Institute of Metabolism and Systems Research, The University of Birmingham, Birmingham, West Midlands, United Kingdom; Instituto Nacional de Geriatria, MEXICO

## Abstract

**Background:**

Frailty and sarcopenia are common in older people and are associated with adverse outcomes including increased mortality and morbidity. It is unclear whether screening for frailty and sarcopenia would identify specific populations most at risk of poor outcomes during unplanned hospital admissions, which screening tools should be used and what the trajectory of both conditions are over the course of an admission. The TYSON study is an observational cohort study aiming to determine the prevalence, trajectory and outcomes associated with frailty and sarcopenia in different patient cohorts. This protocol tests the feasibility and acceptability of TYSON processes.

**Objectives:**

To determine in acutely admitted medical patients who are older adults: Primary: The feasibility and acceptability of frailty and sarcopenia assessments; Secondary: (1) Differences in community and hospital frailty assessments, as assessed by the medical team, the patient and elderly care physicians, (2) The dynamic changes in frailty and sarcopenia during a hospital admission, and patient outcomes; Exploratory: Inflammatory and metabolic mediators associated with frailty and sarcopenia.

**Methods:**

A single centre, prospective observational study including patients aged ≥ 65 years admitted to an acute medical unit. Frailty assessments include the Rockwood clinical frailty and e-frailty index. Sarcopenia assessments include the Bilateral Anterior Thigh Thickness (BATT) measurement. Each participant will be asked to complete 5 visits, at day 0, day 3, day 7, month 3 and month 6. Blood samples will be collected to explore inflammatory and metabolic markers associated with frailty and sarcopenia. The study and protocol have been ethically approved by the Health Research Authority (REC 20/WA/0263).

**Discussion:**

The study will determine the feasibility and acceptability of frailty and sarcopenia assessments in an acute hospital setting, and inform on the prevalence, trajectory and associated outcomes of frailty and sarcopenia in this group of patients. An inflammatory and metabolic profile will be explored in frailty and sarcopenia.

## Introduction

Our global population is ageing. This is associated with increasing demand for health and social care services, especially in older adults who are frail, have sarcopenia, and who have complex care needs.

Frailty is defined as the state of vulnerability resulting in sudden health state changes triggered by relatively minor stressor events [1]. It is associated with adverse effects both out-of-hospital and in-hospital, including poor quality of life, falls, increased use of social services, higher likelihood of institutionalisation, higher hospital length of stay and death [2]. In some circumstances, frailty is reversible, and its impacts can be mitigated through targeted interventions such as Comprehensive Geriatric Assessments (CGA) [3, 4].

There is no internationally agreed gold standard to diagnose frailty. There are a number of assessment tools including (but not limited to) the Clinical Frailty Scale (CFS) [5], the Frailty phenotype (FI) [6], the Hospital Frailty Risk Score (HFRS) [7] or the Edmonton frail scale [8]. A recent systematic review summarised the validated frailty assessment tools globally used in Acute Medical Units and reported significant heterogeneity in terms of approach [9]. Screening for frailty is recommended both in the community [10] and during hospital admissions [11, 12] and there has been increasing interest in cohorting frail patients during acute medical admissions into Acute Frailty Units (AFUs) to improve outcomes [13, 14] although the evidence base to support this practice remains in its infancy.

Sarcopenia is defined by the presence of low muscle strength and low muscle mass. Where there is associated low physical performance, sarcopenia is considered severe [15]. It is highly prevalent in older adults and often co-exists with frailty although it can occur independently. Sarcopenia is also associated with adverse hospital outcomes, including mortality and morbidity [15]. However, as with frailty, it is treatable in many with interventions including resistance training, aerobic exercise, adequate nutrition, and supplements such as vitamin D [16, 17].

Computer tomography (CT) and Magnetic Resonance Imaging (MRI) are gold standards imaging to confirm low muscle mass or quantity [15]. However, these are high costs and burdensome procedures, and other assessments have been used including ultrasound as a novel and non-invasive diagnostic tool for sarcopenia [18–20]. The Bilateral Anterior Thigh Thickness (BATT) protocol is one with excellent correlation with physical parameters of muscle health. It has recently been validated for the assessment and diagnosis of sarcopenia [21].

It is well documented that hospital admission is associated with a loss of muscle mass and function, especially in older adults [22–24]. Some studies support inflammation being a pathological driver of sarcopenia, especially during acute illness. High levels of CRP and IL-6 [25, 26] as well as TNF-∝ [27, 28], were mainly found to be significantly associated with a loss of muscle strength and muscle mass.

Despite recognising the association between frailty and sarcopenia and adverse outcomes during an hospital admission, the impact of routinely screening for frailty and sarcopenia in acute medical units is currently not known. The provision of a CGA for all older adults would have significant workforce implications for Geriatric medicine across healthcare settings. Instead, focusing resource on people either at risk of developing frailty and sarcopenia or with treatable elements to these conditions may be a pragmatic approach to maximise benefit. However, currently it is unclear how to best identify this patient cohort. However, there are a number of tools available to identify both conditions which are in clinical use, and it is unclear how tool selection will impact on case mix. Further, to identify people with treatable elements within both conditions, understanding the trajectory of both conditions during the admission and beyond would be helpful, but few studies map changes in measurements over time [29–31].

The TYSON study aims to assess the prevalence of both frailty and sarcopenia amongst older patients admitted to acute medical units, determine how these conditions change over the course of the admission and assess if it is possible to predict outcomes including recovery to baseline function, in-patient mortality, an increase in social care need and readmission. Furthermore, as inflammation is considered important in both frailty and sarcopenia, this study will explore relationships between (1) inflammatory cytokines (2) metabolic markers and both the degree of sarcopenia and frailty present on admission and their trajectory over time.

However, since it is unclear whether it is possible to perform serial comprehensive assessments of frailty and sarcopenia in acutely unwell older adults, the primary objective of TYSON is the feasibility and acceptability of performing these measures.

### Objectives

The study has primary, secondary and exploratory objectives. These include:

#### Primary objective

To determine the feasibility and acceptability of formal frailty and sarcopenia assessments in acutely admitted medical patients.

#### Secondary objectives

1. To assess differences in how frailty was assessed in community settings by the general practitioner, versus frailty assessments during acute presentation to hospital. This will assess the difference between pre-admission frailty and admission frailty.
2. To assess differences in frailty assessments made by the patient, the acute medical staff, and Geriatric physicians during the acute admission. This will assess differences in frailty assessments conducted by expert clinicians (Geriatricians), general physicians (acute medical staff) and the patient. The Geriatrician assessment will be considered the gold standard.
3. To assess sarcopenia at presentation during the acute admission. This will help determine the prevalence and severity of sarcopenia on admission in an acute medical unit. Understanding this will help plan targeted interventions in order to reverse this condition.
4. To assess the change in frailty and sarcopenia over the acute admission. Serial assessments will help understand the trajectory and variability of both conditions over time, potentially identifying individuals where interventions could be targeted.
5. To determine the relationship between frailty and sarcopenia on admission, their trajectories, and clinically relevant outcomes such as length of stay, adverse hospital events (such as falls and infections), an increase in care requirements and death. This will help establish any relationships between changing trajectories of frailty and sarcopenia and outcomes.

#### Exploratory objective

To assess inflammatory and metabolic mediators and determine the relationships between inflammation, frailty and sarcopenia and poor clinical outcomes in acutely unwell older patients.

## Material and methods

### Study design

TYSON feasibility is a single-centre, prospective observational study of older patients acutely unwell with a medical (non-surgical) health concern and admitted to hospital.

### Study setting

The study will take place in University Hospitals Birmingham NHS Foundation Trust (UHB), with recruiting taking place in the Queen Elizabeth Hospital Birmingham (QEHB). QEHB is a large tertiary hospital, which provides care for over 130,000 acute medical inpatient episodes each year. Patients will be recruited from the Acute Medical Unit.

### Sample size

Mortality is the most commonly cited primary outcome for studies assessing frailty [32–35] and sarcopenia [36–38]. In light of this, we proposed to power our study on 90-day mortality, as this is likely to be the outcome of the full study. At this feasibility stage, we wish to determine the likely timelines needed to recruit sufficient patients to assess this outcome. Previous studies describe an approximate 20% mortality in 90 days for older adults admitted to hospital [39, 40]. Assuming this will be the case for this study, a sample size of 165 will be associated with 33 deaths in 90 days. This will allow us to report an estimated AUROC of 0.70, with alpha of 0.05 and power of 0.90.

### Participants recruitment

The study protocol flowchart, including recruitment process is illustrated in S1 Fig.

#### Inclusion criteria

1. Patients aged 65 and above,
2. Patients within 72 hours of their admission to the Acute Medical Unit
3. Patients with an unplanned medical (non-surgical) admitting complaint.
4. Patients meeting above criteria with or without capacity

#### Exclusion criteria

1. Patients aged less than 65
2. Patients declining consent
3. Personal Consultee, when available, does not provide consent
4. Professional Consultee, if used, does not provide consent
5. Patients expected not to survive for 24 hours and receiving end of life care
6. Patients admitted with a non-medical presenting complaint.

#### Recruitment

Potential participants in this study will be screened for by the members of the direct medical care team. Potential participants will be approached for consent by member of the research team.

#### Consent

All staff will be trained in the ethical principles underpinning informed consent as per good clinical practice, and to assess capacity.

Where the participant has capacity to consent, informed, written consent will be sought.

It is expected that some patients who would be suitable for the study may not have capacity to consent at the time of recruitment due to an altered level of consciousness associated with acute ill health (termed delirium). Delirium is common in older adults during an acute admission, thought to effect up to 15.2% of geriatric patients in emergency department [41]. The exclusion of these patients would bias results and potentially prevent this group of patients from benefiting from evidence-based care pathways.

In such cases, where a patient is deemed not to have capacity, the participant’s personal consultee (namely a relative, friend or partner) will be approached and asked to consider whether the patient would wish to take part in the study. A signed personal consultee declaration form (CDF) is required for study inclusion.

In the event that there is no identifiable personal consultee, a professional consultee will be sought. This will be a senior doctor from the participant’s direct medical care team who is unconnected to the study.

If the participant regains capacity, retrospective consent will be sought during study participation. If the participant does not wish to take part in the study, no further data will be collected. Previously collected data will be included in the study unless the participant specifically requests for this to be removed.

At each visit the participant’s willingness to continue in the study will be ascertained and documented in the medical notes. If the participant lacked capacity at the time of enrolment their capacity will be re-assessed. If the patient still lacks capacity, where possible, continuation in the study will be confirmed by the personal or professional consultee.

The participants’ recruitment for our study started in October 2021.

### Outcomes

#### Primary outcome

Feasibility and acceptability of the study. We will report:

1. The proportion of patients who consented to (or consultee supported) study participation from the total proportion of patients who met the inclusion and exclusion criteria.
2. The proportion of assessments completed at each visit. The healthcare professional or patient-reported reasons for non-completion will be documented and assessed in themes.
3. The proportion of visits completed. The healthcare professional or patient-reported reasons for non-completion will be documented and assessed in themes.

#### Secondary outcomes

1. All causes of mortality at six months (outcome which powered the study)
2. Changes in frailty scores from GP assessment in community (using electronic frailty index (eFI)) to acute presentation (CFS).
3. Differences in Clinical Frailty Scale (CFS) as performed by acute medical team, the patient and formally assessed frailty index (Fried’s FI)
4. The presence and severity of sarcopenia
5. Changes in frailty score at Day 3 and Day 7 and 3 months compared with baseline
6. Changes in sarcopenia at Day 3 and 7 and 3 months compared with baseline
7. Place of discharge and changes in care requirements
8. Length of stay
9. Re-admissions during follow up period

These will be used to help identify the most likely outcome which would be used in a definitive study, with mortality the most likely primary outcome.

#### Exploratory outcome

Inflammatory and metabolic mediators (hsCRP, IL-6, TNF-α, IGF-1, IL-1ß, IL-8, vitamin D) at baseline, Day 3, 7 and 3 months.

### Description of study procedures

As this is a feasibility and acceptability study, although participants will be asked to take part in all study visits and processes, participants may continue in the study with any level of participation. A flowchart of the study activities for each visit is illustrated in S2 Fig.

All participants will have their demographics, presenting symptoms, medical history, medications, smoking and alcohol history, recorded. The participant’s past medical history will be used to derive the Geriatric Index of Comorbidity (GI-C). Observations and blood test results, taken as part of routine care will be collected. See a in S1 Appendix for a full list of information recorded.

#### Frailty assessment

Frailty will be assessed at each visit for all participants using two validated tools, the Frailty Index–FI [6] (b in S1 Appendix), and the Clinical Frailty Scale (CFS) [42] (c in S1 Appendix). Where available, and if calculated within 2 weeks, the electronic–FI (e-FI) [43] will be noted from the primary care record. The CFS [5] will be determined by the acute care admission team, away from the participant and self-selected by the participant.

#### Mini-nutritional assessment

The MNA^®^- SF is a validated assessment tool for nutritional status [44]. Additional information that will be collected will include food intake, specifically protein and fruit or vegetable intake, fluid intake, mid-arm circumference, and calf circumference. The MNA will be measured at each visit for all participants (d in S1 Appendix) and will assess the degree of malnutrition which can be an important risk factor for worse hospital outcomes.

#### Katz activities of daily living (ADLs) and Lawton instrumental ADLs

Katz ADLs provides a measure of ability to carry out basic ADLs that include bathing, dressing, toileting, transferring, continence, and feeding (e in S1 Appendix) [45, 46]. Lawton IADLs measure more complex activities that include ability to use a telephone, shopping, food preparation, housekeeping, laundry, transportation, taking medications, and managing finances (f in S1 Appendix) [47]. These will be collected at the initial visit and the scales will assess the degree of loss of independence attributed to the acute illness that led them to their admission.

#### Handgrip strength measurements

Handgrip strength will be measured at each visit using a handheld dynamometer. Where the participant can sit out in a chair, handgrip strength will be measured with the arm flexed at 90° at the elbow and the forearm supinated. If the participant cannot sit out and measurements are taken in the bed, this will be recorded, and measurements will instead be performed in the most upright position that is feasible. Participants will be asked to “squeeze as hard as they can”. Handgrip strength will be measured at each visit, twice on each side and the best recording of all will be used for analysis (g in S1 Appendix).

#### Four metres walk speed test

This test will be repeated as described [48] at each visit, twice: first, participant will be advised to walk comfortably at usual pace for the entire distance and the best time will be recorded. Assisted devices, if used, will be documented for each test and during each visit (h in S1 Appendix).

#### Bilateral anterior thigh thickness (BATT) measurements

The BATT protocol will be used as described [21] for muscle mass measurements and all ultrasound measurements will be taken in line horizontally with these marks (i in S1 Appendix) [49]. The intra-rater and inter-rater reliability of BATT has been previously shown to be excellent when using the same protocol and same machine. Images will be saved and downloaded for assessment. The diagnosis and severity of sarcopenia will be determined using the EWGSOP2 criteria [15].

#### Blood sampling

Blood samples will be taken peripherally using the BD vacutainer^®^ Safety-Lok^™^ system in sterile vacutainers (BD biosciences, California-USA) by trained staff. Participants will have 20mL of whole blood drawn at each visit. Serum and plasma will be prepared using standardised techniques [50], aliquoted into 1mL samples and stored at -80°C, labelled with the participants unique study number, date and aliquot number.

#### Laboratory tests

IL-6, IL-8, TNF-α, IL-1ß and hsCRP ELISAs (Thermo Fisher Scientific plates) will be performed on serum samples (100 μl, 100 μl, 100 μl, 200 μl and 10 μl respectively) while IGF-1 monoclonal antibodies ELISAs and Vitamin D will be performed on plasma samples (50 μl and 200 μl). The tests will be conducted according to the manufacturer’s protocols (ebioscience, UK).

Additional biomarkers may be tested as part of this project as appropriate.

### Study schedule

To be included in the study, participants will be recruited within 72 hours of their initial presentation to hospital. Study visits (including their timings) and processes at each visit are outlined in the flowchart of study activities.

### Data collection and management plan

Data will be collected on case report forms (CRFs) designed by the research team, which will be filed in the investigator site file (ISF). Each participant will be given their own unique study identifier for the purposes of this study.

### Data storage

Pseudonymised participant data will be held in a purposefully designed database. The database will be stored on the sponsor server which is backed up every twenty-four hours. Access will be restricted by password, accessible only to members of the research team, the sponsor, and any regulatory bodies. Access will be auditable. The data will be locked prior to analysis. Data will be stored for ten (10) years in line with The University of Birmingham guidelines. Data will be stored as per the Data Protection Act, 2018.

### Statistical analysis

Data analysis will be conducted using IBM SPSS^®^ Version 22. Outcomes will be summarised at baseline, day 3 of admission, day 7 of admission, and month 3 post-discharge.

1. To determine feasibility and acceptability, we will analyse data using descriptive and where applicable, qualitative statistics.
2. To assess the differences in the scores for our secondary objectives, we will use a combination of traditional statistical techniques, including T-tests and non-parametric equivalents.
3. To calculate mortality, we will determine area under the receiving operating curves (AUROC).

The following models will be used:

1. Unadjusted model with all-cause mortality at 12 months as the outcome of interest, and the secondary outcome as the covariate of interest (i.e., change in CFS score, BATT, handgrip strength and/or gait speed from baseline to 7 days)
2. Adjusted model with all-cause mortality at 12 months as the outcome of interest, and the secondary outcome as the covariate of interest (i.e., change in CFS score, BATT, handgrip strength and/or gait speed from baseline to 7 days), with adjustment for baseline CFS score and patient demographics (e.g. age, gender, ethnicity, etc).
3. Model with all-cause mortality at 12 months as the outcome of interest, with adjustment for baseline CFS and all secondary outcomes as covariates to establish the strength of association between those secondary outcomes and the primary outcome.

### Ethical considerations

#### Assessment and management of risk

The study is very low risk to the participant and uses assessments and interventions techniques, considered safe for the older adults. All procedures will be completed by trained investigators.

Venepuncture is associated with slight discomfort and bruises. Whenever possible, blood will be taken alongside samples collected as part of participants’ routine care.

When performing test of mobility (4 metres walk speed test), the investigator will be responsible for first assessing that the environment is safe (e.g. no obstacles present, the participant is wearing appropriate footwear and sensory aids if necessary, walking aids are provided if needed). If either the investigator or the participant do not feel safe to perform the test, then they will not proceed to do so, and this will be documented on the CRF. If the participant does fall, then the investigator will abide by local policy for manual handling and care of the falling patient. This includes arranging review within the emergency department and contacting the GP to notify them of the fall.

The participants may be diagnosed with previously unknown diseases during the process of research data collection. These will be explained to the participant, including the potential consequences and further investigations or treatment that might be indicated. These findings will be relayed to the participants’ General Practitioners (GP) or secondary care medical team, who will be advised to arrange further management or to refer to appropriate specialties; this is usual practice in the United Kingdom (UK). Specific consent will be gained from the participant to inform the GP of any relevant results and study participation.

### Patient & public involvement (PPI)

We have discussed this study with older people who have been admitted to the AMU in QEHB and their relatives. This includes the concept of frailty and sarcopenia which was considered important by older people, the tests included in the study, the blood tests; the need to contact the GP and for the follow up visit. Results of our study will be presented at public events, annual conferences, workshops, webinars and will be published in hospital journals or newsletters and scientific journals. All involvement will happen according to INVOLVE guidelines: http://www.invo.org.uk/.

### Status and timeline of the study

Participant recruitment started in October 2021, although this was initially paused due to the COVID-19 pandemic. Recruitment should be completed by November 2023.

## Discussion

This study aims to test the feasibility and acceptability of serial assessments of sarcopenia and frailty in older adults acutely admitted to hospital with a medical condition. It will also determine the prevalence of frailty and sarcopenia in older patients acutely admitted in hospital through acute medical department, compare self-reported and healthcare professional assessments of frailty, and the trajectory of both conditions over the course of the study. It will be one of the most comprehensive assessments of sarcopenia and frailty attempted within acute medical units and should begin to inform how to select patients for a CGA. The inflammatory cytokines and/or metabolic markers may provide insight into pathological mechanisms driving frailty and sarcopenia progression or resolution.

The study benefits form the inclusion of validated assessment tools, and being based in Birmingham, UK, will recruit for a diverse population (with 40% of residents self-defining as from BAME communities). As this is a feasibility study primarily, it is unclear how complete data capture will be. A potential limitation is the single centre design, although this would be addressed were the feasibility study successful.

The results will be presented at in peer review journals and scientific conferences. Results will also be shared through a series of public engagement events including webinars.

The anonymised participant level dataset will not be publicly available but will be available from the principal investigator upon reasonable request.

Amendments to the protocol, study design or supporting documents will be approved by the sponsor, HRA and REC prior to implementation. All documents will be maintained with version number and tracked changes.

### Authorship eligibility guidelines and any intended use of professional writers

All investigators and collaborators who have had significant input into the development of this protocol and conduct of this research will be granted single (rather than group) authorship on publications arising from this study. Except in the case of additional sub-studies, the first author will normally be the principal investigator, and the last author will be the chief investigator.

## Supporting information

S1 Appendix The TYSON study protocol: Appendixes. (DOCX)

S1 Fig The TYSON study protocol flowchart. (TIF)

S2 Fig The TYSON study’s activities flowchart. (TIF)
