# Supplementary material for: Exploring fraity and sarcopenia in older adults admitted to acute medical unit, looking at prevalence, trajectory, and outcomes: A protocol testing the feasibility and acceptability of the TYSON study
Source: PLoS One. 2023 Nov 3;18(11):e0293650. doi: 10.1371/journal.pone.0293650 (PMC10624263; doi:10.1371/journal.pone.0293650)
Supplement: S1 Appendix — (DOCX) [file pone.0293650.s001.docx]

**The TYSON study protocol: Appendixes.**

1. Case report form: History taking.

Initials: Date: Consent

DOB: Age: Gender:

**PAST MEDICAL HISTORY**

| Stroke | Y / N |
| --- | --- |
| MI | Y / N |
| CCF | Y / N |
| DM | Y / N |
| COPD | Y / N |
| Cancer | Y / N |

| Ethnicity |  | Using ONS guidelines |
| --- | --- | --- |
| Smoker | Y / N / Ex |  |
| Pack years |  | 1 pack year = year where 20 cigarettes are smoked everyday |
| Alcohol consumption |  | Average units per week |
| Occupation |  | If multiple choose occupation of longest time-period |
| Education |  | Years of full-time education |

Comorbidities: “Any other health conditions not mentioned above?”

| Cardio-metabolic cluster |  | Psych-neuro Cluster |  | Musculoskeletal Cluster |  |
| --- | --- | --- | --- | --- | --- |

Self-reported health: “How do you think your health is on a scale of 1-5, 1 being poor and 5 being good? “

1 2 3 4 5

Falls within the last year: 1 2 3 4 5 5+

Continence: “Do you have a problem with losing control of urine when you don’t want to?”

Y N

Weight loss:

“In the last year have you lost more than 10 pounds (4.5kg) unintentionally or is your clothing looser?”:

Y N

“And over the last 3 months have you lost more than half a stone (3kg)?”:

>3kg = 0, Don’t know = 1, 1-3kg = 2, None = 3

No of Medications

**DRUG HISTORY**

*On regular prescription including as required medications*

**SOCIAL HISTORY**

Lives: Alone / Family / Partner / Friend

Have you got family or friends that in times of need you can rely on: Y / N

Place of Residence: House / Flat /Bungalow / Warden Controlled Flat /RH / NH

Walking Aids Indoors: I / S / 3 wheels / RF Outdoors: I / S / 3 wheels / RF

*I = Independent, S = Stick, 3 wheels = 3 wheelers, RF = rollator frame*

1. Phenotypic diagnosis of frailty (criteria Fried scale)

| Criteria | Criteria to score positive | | Source |
| --- | --- | --- | --- |
| Handgrip strength (kg) | Male | Female | Original study 2001 |
|  | BMI <= 24: <=29  BMI 24-26: <=30  BMI 26-28: <=30  BMI >28: <=32 | BMI <=24: <=17  BMI 24-26: <=17.3  BMI 26-28: <=18  BMI >28: <=21 |  |
| Gait speed (m/s) | Male | Female | Original study 2001 |
|  | <=1.73m height: <=0.65  >1.73m height: <=0.76 | <= 1.59m height: <= 0.65  >1.59m height: <=0.76 |  |
| Self-reported exhaustion | Answers “most of the time” or “all of the time” to the following questions.  How often over the last week have you felt that the following statements were true?   - “I felt that everything I did was an effort” - “I could not get going” | | Original study 2001 |
| Weight loss | 4.5kg OR 5% total body weight loss over last year | | Original study 2001 |
| Physical activity | Score if any of the following are true. In the last month have you:   - Performed no weight bearing physical activity - Spent more than 4 hours/ day sitting - Been for a short walk once/ month or less | | Frailty Intervention Trial (34) |

1. Clinical Frailty Scale (CFS)

| CFS score | Description |
| --- | --- |
| 1  Very fit | Robust, active, energetic and motivated; commonly exercise regularly |
| 2  Fit | No active disease symptoms but less fit than above; exercise often or active occasionally e.g. seasonally |
| 3  Managing well | Medical problems are well controlled but not regularly active beyond routine walking |
| 4  Vulnerable | Not dependent on others for daily help but often symptoms limit activities; common complaint is being “slowed-up” or tired during the day |
| 5  Mildly frail | More evident slowing; need help with high order IADLs; typically, progressively impairs shopping and walking outside alone, meal preparation and housework |
| 6  Moderately frail | Need help with all outside activities; often have problems with stairs and need help with bathing and might need minimal assistance with dressing |
| 7  Severely frail | Completely dependent for personal care; seem stable and not at high risk of dying within 6 months |
| 8  Very severely frail | Completely dependent, approaching the end of life; typically, could not recover from a minor illness |
| 9  Terminally ill | Life expectancy < 6 months but not otherwise evidently frail |

1. Mini-nutritional assessment (MNA)

|  | Options | | Points |
| --- | --- | --- | --- |
| Has food intake declined over the past 3 months due to loss of appetite, digestive problems, chewing or swallowing difficulties? | Severe decrease | | 0 |
|  | Moderate decrease | | 1 |
|  | No decrease | | 2 |
| Weight loss during the last 3 months | > 3kg | | 0 |
|  | Does not know | | 1 |
|  | 1-3kg | | 2 |
|  | No weight loss | | 3 |
| Mobility | Bed or chair bound | | 0 |
|  | Able to get out of bed/ chair but does not go out | | 1 |
|  | Goes out | | 2 |
| Have you suffered psychological stress or acute disease in the past 3 months? | Yes | | 0 |
|  | No | | 2 |
| Neuropsychological problems | Severe dementia or depression | | 0 |
|  | Mild dementia | | 1 |
|  | No psychological problems | | 2 |
| Body Mass Index (BMI) | < 19 | | 0 |
|  | 19 to less than 21 | | 1 |
|  | 21 to less than 23 | | 2 |
|  | >= 23 | | 3 |
| Lives independently (not in a care home) | Yes | | 1 |
|  | No | | 0 |
| Takes more than 3 prescription drugs per day | No | | 0 |
|  | Yes | | 1 |
| Pressure sores or skin ulcers | No | | 0 |
|  | Yes | | 1 |
| How many full meals does the patient eat daily? | 1 meal | | 0 |
|  | 2 meals | | 1 |
|  | 3 meals | | 2 |
| Selected consumption markers for protein intake | 1. At least one serving of dairy products (milk, cheese, yoghurt) per day  2. Two or more servings of legumes or eggs per week  3. Meat, fish, or poultry every day | If 0 or 1 yes | 0.0 |
|  |  | If 2 yes | 0.5 |
|  |  | If 3 yes | 1.0 |
| Consumes two or more servings of fruit or vegetables per day? | No | | 0 |
|  | Yes | | 1 |
| How much fluid is consumed per day? | Less than 3 cups | | 0.0 |
|  | 3 to 5 cups | | 0.5 |
|  | More than 5 cups | | 1.0 |
| Mode of feeding | Unable to eat without assistance | | 0 |
|  | Self-fed with some difficulty | | 1 |
|  | Self-fed without any problem | | 2 |
| Self-view of nutritional status | Views self as being malnourished | | 0 |
|  | Is uncertain of nutritional state | | 1 |
|  | Views self as having no nutritional problem | | 2 |
| In comparison with other people of the same age, how does the patient consider his/her health status? | Not as good | | 0.0 |
|  | Does not know | | 0.5 |
|  | As good | | 1.0 |
|  | Better | | 2.0 |
| Mid-arm circumference (MAC) in cm | MAC less than 21 | | 0.0 |
|  | MAC 21 to 22 | | 0.5 |
|  | MAC greater than 22 | | 1.0 |
| Calf-circumference (CC) in cm | CC less than 31 | | 0 |
|  | CC 31 or greater | | 1 |

1. Katz basic ADLs

Please tick the most relevant response for each category:

| Activities | Independent (1 point) | Dependent (0 point) |
| --- | --- | --- |
| Bathing | Bathes self completely or needs help in bathing only a single part of the body such as the back, genital area or disabled extremity. | Needs help with bathing more than one part of the body, getting in or out of the tub or shower. Requires total bathing. |
| Dressing | Gets clothes from closets and drawers and puts on clothes and outer garments complete with fasteners. May have help tying shoes. | Needs help with dressing self or needs to be completely dressed. |
| Toileting | Goes to toilet, gets on and off, arranges clothes, cleans genital area without help. | Needs help transferring to the toilet, cleaning self or uses bedpan or commode. |
| Transferring | Moves in and out of bed or chair unassisted. Mechanical transferring aides are acceptable. | Needs help in moving from bed to chair or requires a complete transfer. |
| Continence | Exercises complete self-control over urination and defecation. | Is partially or totally incontinent of bowel or bladder. |
| Feeding | Gets food from plate into mouth without help. Preparation of food may be done by another person. | Needs partial or total help with feeding or requires parenteral feeding. |

1. Lawton IADLs

Please select the most appropriate option for each category by circling the relevant points score on the far-right column:

| Activities | Options | Points |
| --- | --- | --- |
| Ability to use telephone | 1. Operates telephone on own initiative – looks up and dials numbers etc. | 1 |
|  | 1. Dials a few well-known numbers | 1 |
|  | 1. Answers telephone but does not dial | 1 |
|  | 1. Does not use telephone at all | 0 |
| Shopping | 1. Takes care of all shopping needs independently | 1 |
|  | 1. Shops independently for small purchases | 0 |
|  | 1. Needs to be accompanied on any shopping trip | 0 |
|  | 1. Completely unable to shop | 0 |
| Food preparation | 1. Plans, prepares, and serves adequate meals independently | 1 |
|  | 1. Prepares adequate meals if supplied with ingredients | 0 |
|  | 1. Heats and serves prepared meals, or prepares meals but does not maintain adequate diet | 0 |
|  | 1. Needs to have meals prepared and served | 0 |
| Housekeeping | 1. Maintains house alone or with occasional assistance e.g. help with “heavy-work” | 1 |
|  | 1. Performs light daily tasks such as dishwashing, bedmaking | 1 |
|  | 1. Performs light daily tasks but cannot maintain acceptable level of cleanliness | 1 |
|  | 1. Needs help with all home maintenance tasks | 1 |
|  | 1. Does not participate in any housekeeping tasks | 0 |
| Laundry | 1. Does personal laundry completely | 1 |
|  | 1. Launders small items e.g., rinses socks, stockings etc. | 1 |
|  | 1. All laundry must be done by others | 0 |
| Mode of transportation | 1. Travels independently on public transport or drives own car | 1 |
|  | 1. Arranges own travel via taxi, but does not otherwise use public transportation | 1 |
|  | 1. Travels on public transport when assisted or accompanied by another | 1 |
|  | 1. Travel limited to taxi or automobile with assistance of another | 0 |
|  | 1. Does not travel at all | 0 |
| Responsibility for own medications | 1. Is responsible for taking medication in correct dosages at correct time | 1 |
|  | 1. Takes responsibility if medication is prepared in advance in separate dosages | 0 |
|  | 1. Is not capable of dispensing own medications | 0 |
| Ability to handle finances | 1. Manages financial matters independently (budgets, writes checks, pays rent, bills, goes to bank); collects and keeps track of income | 1 |
|  | 1. Manages day-to-day purchases but needs help with banking, major purchases etc. | 1 |
|  | 1. Incapable of handling money | 0 |

1. Handgrip strength measurement

Dominant:

Non-dominant:

*Ask participant to sit down with feet flat on the floor. Hold the dynamometer in their hand with upper arm by their side and forearm at 90^O^ angle with wrist straight and un-rotated. Support the dynamometer. Complete twice for each side and record the highest value.*

1. Four metres walk speed

4 metres walk course: _________________________________________________________________

*On a measured 6 metres walk course (2 meters to get up to speed and 4 metres for measuring walk speed) ask the participant to walk at normal speed and time how long it takes them to walk 4 metres. Allow them 2 metres to get up to speed and start the clock when they cross the 2 metres line. Stop the clock at 6 metres line.*

1. BATT ultrasound measurements report form

|  | Right | | | | Left | | | |
| --- | --- | --- | --- | --- | --- | --- | --- | --- |
| Rectus Femoris |  |  |  |  |  |  |  |  |
| Vastus Intermedius |  |  |  |  |  |  |  |  |
| Subcutaneous |  |  |  |  |  |  |  |  |

Height:

Weight:

Abdominal circumference:

Hip circumference:

1. Psychological assessment

Please circle the most appropriate answer for each question.

| Are you basically satisfied with your life? | Yes / **No** |
| --- | --- |
| Do you feel that your life is empty? | **Yes** / No |
| Are you afraid something bad is going to happen to you? | **Yes** / No |
| Do you feel happy most of the time? | Yes / **No** |

1. Stroop colour / word test

| Word |  |
| --- | --- |
| Colour |  |
