# Supplementary figures and images for: Exploring fraity and sarcopenia in older adults admitted to acute medical unit, looking at prevalence, trajectory, and outcomes: A protocol testing the feasibility and acceptability of the TYSON study
Source: PLoS One. 2023 Nov 3;18(11):e0293650. doi: 10.1371/journal.pone.0293650 (PMC10624263; doi:10.1371/journal.pone.0293650)

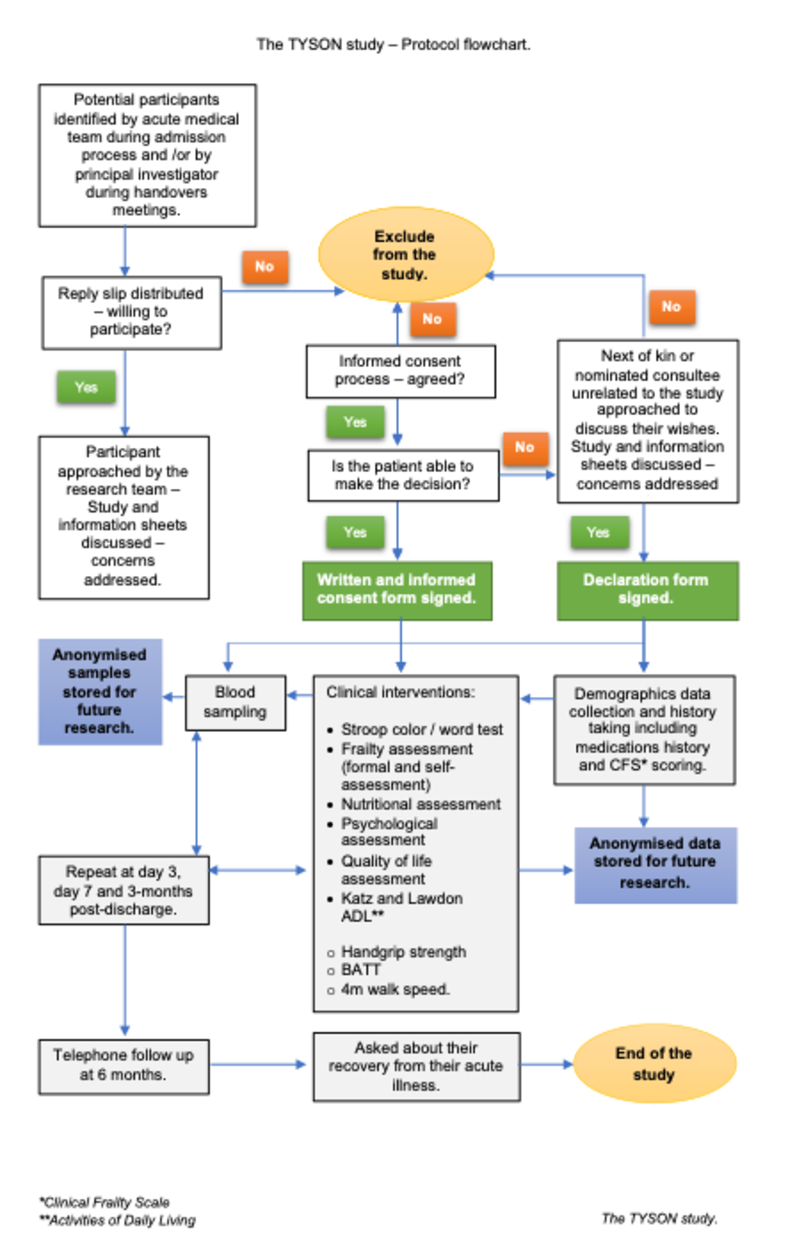

Supplement: S1 Fig — (TIF) [file pone.0293650.s002.tif]

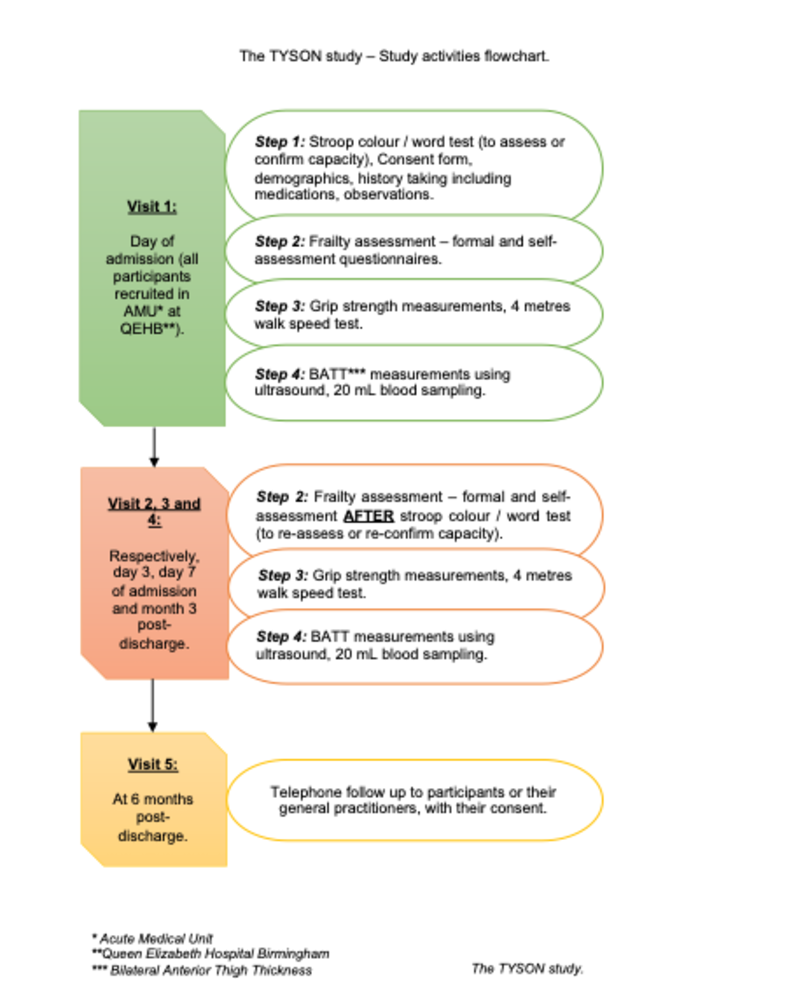

Supplement: S2 Fig — (TIF) [file pone.0293650.s003.tif]
